# The MicroRNA Prediction Models as Ancillary Diagnosis Biomarkers for Urothelial Carcinoma in Patients With Chronic Kidney Disease

**DOI:** 10.3389/fmed.2021.726214

**Published:** 2021-10-01

**Authors:** An-Lun Li, Che-Yi Chou, Chien-Lung Chen, Kun-Lin Wu, Shih-Chieh Lin, Hung-Chun Chen, Ming-Cheng Wang, Chia-Chu Chang, Bang-Gee Hsu, Mai-Szu Wu, Nianhan Ma, Chiu-Ching Huang

**Affiliations:** ^1^Department of Biomedical Sciences and Engineering, National Central University, Taoyuan, Taiwan; ^2^Division of Nephrology, Department of Internal Medicine, Asia University Hospital, Taichung, Taiwan; ^3^Department of Nephrology, Landseed International Hospital, Taoyuan, Taiwan; ^4^Division of Nephrology, Department of Internal Medicine, Taoyuan Armed Forces General Hospital, Taoyuan, Taiwan; ^5^Institute of Basic Medical Sciences, College of Medicine, National Cheng Kung University, Tainan, Taiwan; ^6^Division of Nephrology, Kaohsiung Medical University, Kaohsiung, Taiwan; ^7^Division of Nephrology, Cheng Kung University Hospital, Tainan, Taiwan; ^8^Division of Nephrology, Department of Internal Medicine, Kuang Tien General Hospital, Taichung, Taiwan; ^9^Department of Nutrition, Hungkuang University, Taichung, Taiwan; ^10^Division of Nephrology, Buddhist Tzu Chi General Hospital, Hualien, Taiwan; ^11^Division of Nephrology, Taipei Medical University and Hospitals, Taipei, Taiwan; ^12^Division of Nephrology and The Kidney Institute, China Medical University and Hospitals, Taichung, Taiwan

**Keywords:** microRNA (miRNA), urothelial carcinoma (UC), chronic kidney disease, biomarker, biofluid

## Abstract

Urothelial carcinoma is a common urological cancer in chronic kidney disease patients. Cystoscopy and urine cytology are the clinical diagnostic tools for UC. However, cystoscopy is an invasive procedure, while urine cytology showed low sensitivity for low-grade urothelial tumors. High accuracy with non-invasive tools for UC is needed for CKD patients. Our study collected a total of 272 urine and 138 plasma samples to detect the miRNA expression levels for establishing UC signatures from CKD patients. Seventeen candidate miRNAs of biofluids were selected and confirmed by qRT-PCR. Our results showed that urinary miR-1274a and miR-30a-5p expression levels were significantly lower but miR-19a-5p expression levels were higher in UC when compared with CKD. In plasma samples, miR-155-5p, miR-19b-1-5p, miR-378, and miR-636 showed significantly lower expression in UC compared to those with CKD. The Kaplan-Meier curve showed that lower expression of miR-19a, miR-19b, miR-636 and miR-378, and higher expression of miR-708-5p were associated with poor prognosis in patients with bladder cancer. In addition, we produced classifiers for predicting UC by multiple logistic regression. The urine signature was developed with four miRNAs, and the AUC was 0.8211. Eight miRNA expression levels from both urine and plasma samples were examined, and the AUC was 0.8595. Two miRNA classifiers and the nomograms could improve the drawbacks of current UC biomarker screenings for patients with CKD.

## Introduction

Urothelial carcinoma (UC) includes bladder cancer and urinary tract cancer. A worldwide report revealed ~549,393 newly diagnosed cases and 199,922 deaths from UC in 2018 (1). UC accounts for 90% of bladder cancer and is the most common malignancy involving the urinary tract (2). UC is responsible for 31% of urinary tract cancers in Taiwan (3). 51–58.6% of UC patients have chronic kidney disease (CKD) and patients with CKD are more at risk of UC (3–6). Advanced stages of CKD are associated with poor prognosis for UC treatment (7, 8). Painless hematuria is the most common presenting symptom in UC, but painless hematuria is also common in patients with CKD. The sensitivity and specificity of UC protein markers are decreased because the serum protein levels are increased in patients with CKD (9–13). Intravenous pyelography or urography cannot be performed in patients with CKD because of the exposure of contrast media (14). The high specificity of urinary cytology can be interfered by the presence of CKD (15). Invasive cystoscopy or ureteroscopy are usually needed to confirm the diagnosis of UC. In addition, the cost of cystoscopy or ureteroscopy is expensive, the procedure is invasive and uncomfortable, and patients need to experience the risk of anesthesia and surgery. Therefore, developing highly accurate non-invasive biomarkers for UC is urgently needed for patients with CKD.

The miRNA pattern in biofluids was thought to provide disease molecular markers to predict or differentiate different types of cancers because the development of cancer is associated with the expression levels of circulating miRNAs (16–18). In addition, miRNAs can be packed and released through exosomes or extracellular vesicles, enhancing their stability in biofluids such as urine and plasma. Some reports have discussed the difference in miRNA expression in biofluids for predicting urological tumors, but most of the studies compared healthy donors with patients with cancer (16, 19–23). Our previous study demonstrated that the miRNA classifier of plasma predicted UC in patients with ESRD (24). In the present work, we investigated the expression levels of miRNAs in the urine and plasma of patients with CKD. We further used these miRNA signatures to develop prediction models of UC for patients with CKD.

## Materials and Methods

### Patients and Samples

The Taiwan Urothelial Cancer Consortium (TUCC) organized a multicenter study of urothelial cancer (UC) from ten hospitals in Taiwan. The ten hospitals are distributed throughout the country (13) (Supplementary Table 4). A total of 272 patients (50, 111, and 111 samples were healthy, CKD and CKD + UC, respectively) participated in this study. The urine and blood samples were collected from control patients after obtaining informed consents. The urine and blood samples were collected from CKD+UC patients within 3 days before the surgery. Samples were centrifuged at 1,700 and 2,000 × g for 20 min. The supernatant was collected and stored at −80°C.

### Ethics Approval and Informed Consent

This study was approved by the internal review board (IRB) of China Medical University Hospital (CMUH 102-REC2- 043) and the IRB of each hospital. Written informed consent was obtained from all patients to use their urine and blood samples. All methods were followed in accordance with guidelines and regulations.

### Total RNA Isolation From Biofluids and miRNA Quantification by RT-PCR

Total RNA from urine and plasma was extracted using TRIzol® LS Reagent and a mirVana™ miRNA Isolation Kit according to the standard protocol. The spiked-in control of cel-miR-39-3p for technical variability followed the previously described (24). The RNA quality was detected by a spectrophotometer (BioTek Instruments, Take3 microplate). The ratios of absorbance 260 nm to the absorbance at 280 or 230 nm have been used as the reference of the purity of RNA samples (A_260_/A_280_ ≒ 2, A_260_/A_230_ ≒ 2-2.2). All RNA samples were stored at −80°C. The TaqMan™ MicroRNA Reverse Transcription Kit (Applied Biosystems) was used to produce the cDNAs from miRNAs, and the standard protocol or ingredients were followed as described previously (25). The microRNA profiling was generated using TaqMan® 2x Universal PCR master mix without UNG and TaqMan® Array Human MicroRNA Cards (4444913). TaqMan® miRNA assays quantified the specific miRNAs expression (4427975) (Thermo Fisher Scientific).

### Data Statistical Analysis

The expression of miRNAs was determined using the 2^−ΔCT^ method relative to RNU6. The miRNA expression data were transformed to the log_10_ form to fit a normal distribution. The value of no detection of miRNA expression was replaced with the −4.5 value in the log_10_ form. Clinical characteristics between healthy, CKD and CKD+UC patients were evaluated using Pearson's chi-squared test for each variable. Normality and Student's *t*-test were used for unpaired comparisons of two groups. All tests were two-tailed and were assessed by Levene's test. All statistical analyses were completed with GraphPad Prism software. Logistic regression of miRNA expression was combined with SigmaPlot software. All statistic methods or procedures were followed as described previously (24).

### Survival Curve Analysis

A KM-plotter analysis was performed to integrate the miRNA expression and survival data from TCGA, GEO and EGA database (http://kmplot.com/analysis/index.php?p=background). miRNA expression values from clinical specimens were used to perform Kaplan-Meier survival curve analysis according to the clinical parameters provided. High and low expression groups were created using an automatic cutoff as described previously (25). The miRNAs expression associated with UC in multivariable logistic regression was used to generate a nomogram for UC. The coding packages of RMS in R software were used to develop the nomogram of UC.

## Results

### Differentially Expressed Urine and Plasma miRNAs Between CKD and CKD + UC

In order to discover an ancillary diagnostic tool for UC in patients with CKD, all samples were collected from ten hospitals throughout Taiwan from 2013 to 2018. We matched the patients with CKD and CKD+UC by sex, age, and CKD stage to select the difference in miRNA expression levels in this study (Table 1). For CKD+UC, blood and urine samples were collected within 3 days before surgery. For the control group, blood and urine samples were collected after tracking their renal functions as CKD. Next, the high throughput and quantitative real-time miRNA PCR array including 754 miRNAs were utilized to detect 22 (11 CKD and 11 CKD+UC) and 16 (8 CKD and 8 CKD+UC) samples of urine and plasma, respectively (Supplementary Table 7). We not only calculated the relative expression levels by RNU6 but also calculated the miRNA ratio of two different miRNAs expression to remove the normalization problem in cell-free biofluids. To date, no literature has noted that any miRNA is a competent internal control in biofluids, and we found that the ratio value method could reduce individual sample differences. We compared miRNA expressions between CKD and CKD + UC samples, and 17 candidate miRNAs were selected from screen set (Table 2).

**Table 1:** Distribution of the clinical status of patients in this study.

		**Screening (*****n*** **=** **22)**							**Training (*****n*** **=** **100)**							**Testing (*****n*** **=** **100)**							**Testing (*****n*** **=** **50)**		
		**CKD**			**UC**				**CKD**			**UC**				**CKD**			**UC**				**Normal**		
		***n***	**Mean**	**SD**	***n***	**Mean**	**SD**	***P*-value**	***n***	**Mean**	**SD**	***n***	**Mean**	**SD**	***P*-value**	***n***	**Mean**	**SD**	***n***	**Mean**	**SD**	***P*-value**	***n***	**Mean**	**SD**
Age		11	66.82	11.25	11	64.45	9.50	0.600^a^	50	65.4	10.6	50	65.78	11.49	0.849^a^	50	61.12	11.22	50	67.44	10.95	0.005^a^	50	61.88	14.52
Sex	F	2			2			1^b^	15			13			0.656^b^	20			18			0.591^b^	18		
	M	9			9				35			37				30			32				32		
Grade	-	11							50			5				50			14				50		
	Low								0			13							9						
	High				11				0			32							27						
CKD stage	0																						50		
	I	1			1			1^b^	9			11			0.856^b^	10			7			0.680^b^			
	II	1			1				10			11				10			10						
	III	3			3				15			14				11			18						
	IV	2			2				10			11				10			7						
	V	4			4				6			3				9			8						

*Each group was well matched for age, sex and CKD stage. n, number in each group; Mean, average of each group; SD, standard deviation; CKD, chronic kidney disease; CKD + UC, the urothelial carcinoma patients with CKD*.

a *Independent samples test*.

b *Pearson chi-square test*.

**Table 2:** miRNA names and sequences.

**miRNA name**	**Mature miRNA sequence**
hsa-miR-586	UAUGCAUUGUAUUUUUAGGUCC
hsa-miR-129-5p	CUUUUUGCGGUCUGGGCUUGC
hsa-miR-33b-5p	GUGCAUUGCUGUUGCAUUGC
hsa-miR-30a-5p	UGUAAACAUCCUCGACUGGAAG
hsa-miR-1274A	GUCCCUGUUCAGGCGCCA
hsa-miR-126-3p	UCGUACCGUGAGUAAUAAUGCG
hsa-miR-210-3p	CUGUGCGUGUGACAGCGGCUGA
hsa-miR-202-3p	AGAGGUAUAGGGCAUGGGAA
hsa-miR-19a-5p	AGUUUUGCAUAGUUGCACUACA
hsa-miR-708-5p	AAGGAGCUUACAAUCUAGCUGGG
hsa-miR-19b-1-5p	AGUUUUGCAGGUUUGCAUCCAGC
hsa-miR-183-3p	GUGAAUUACCGAAGGGCCAUAA
hsa-miR-636	UGUGCUUGCUCGUCCCGCCCGCA
hsa-miR-155-5p	UUAAUGCUAAUCGUGAUAGGGGU
hsa-miR-378	ACUGGACUUGGAGUCAGAAGG
hsa-miR-487a-3p	AAUCAUACAGGGACAUCCAGUU
hsa-miR-150-5p	UCUCCCAACCCUUGUACCAGUG

**Figure 1:**
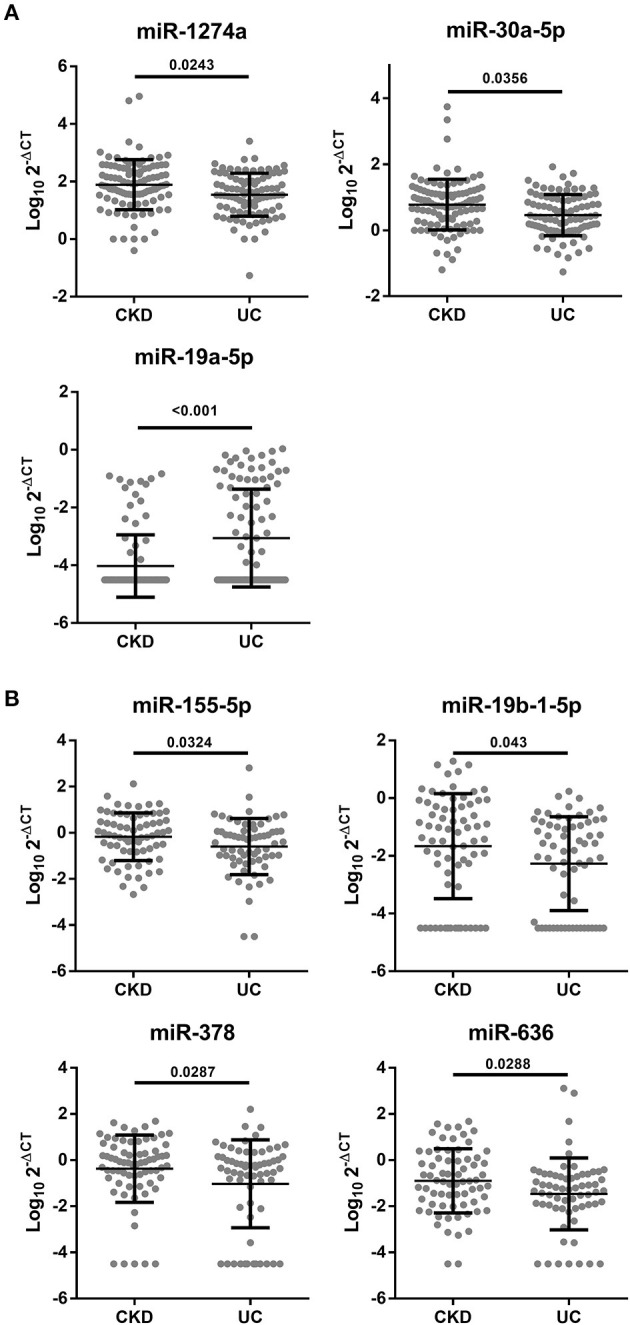
Significant different miRNA expressions in the urine or plasma between CKD and CKD + UC. **(A)** miRNA levels from the urine of patients detected by qRT-PCR using RNU6 as a control (*n* = 200). **(B)** miRNA levels from the plasma were detected by qRT-PCR using RNU6 as a control (*n* = 134). The Y axis presents the expression level (Log10 2-ΔCT). CKD, patients with chronic kidney disease. CKD+UC, the urothelial carcinoma patients with CKD. The *p*-value was analyzed by Student's *t*-test for each miRNA.

Next, we validated the expression levels of 17 candidate miRNAs from a screening set by the single qRT-PCR method and measured 200 urine samples (100 CKD and 100 CKD + UC) and 138 plasma samples (74 CKD and 64 CKD + UC) in training and testing set (Supplementary Table 6). Our results showed that the expression of seven miRNAs was significantly different between the CKD and CKD + UC samples (Figure 1). In urine samples, miR-1274a and miR-30a-5p expression levels were significantly decreased (*p* = 0.0243 and 0.0356, respectively), but the miR-19a-5p expression level was significantly increased (*p* < 0.001) (Figure 1A). In the plasma samples, miR-155-5p, miR-19b-1-5p, miR-378 and miR-636 expression levels were significantly decreased (*p* = 0.0324, 0.043, 0.287, and 0.0288, respectively) (Figure 1B). Interestingly, previous study has shown that miR-30a-5p had significantly low expression levels in plasma samples of patients with BC (26). In addition, miR-155-5p expression was also reported to be significantly decreased in the urine sediment cells of patients with BC (27).

**Figure 2:**
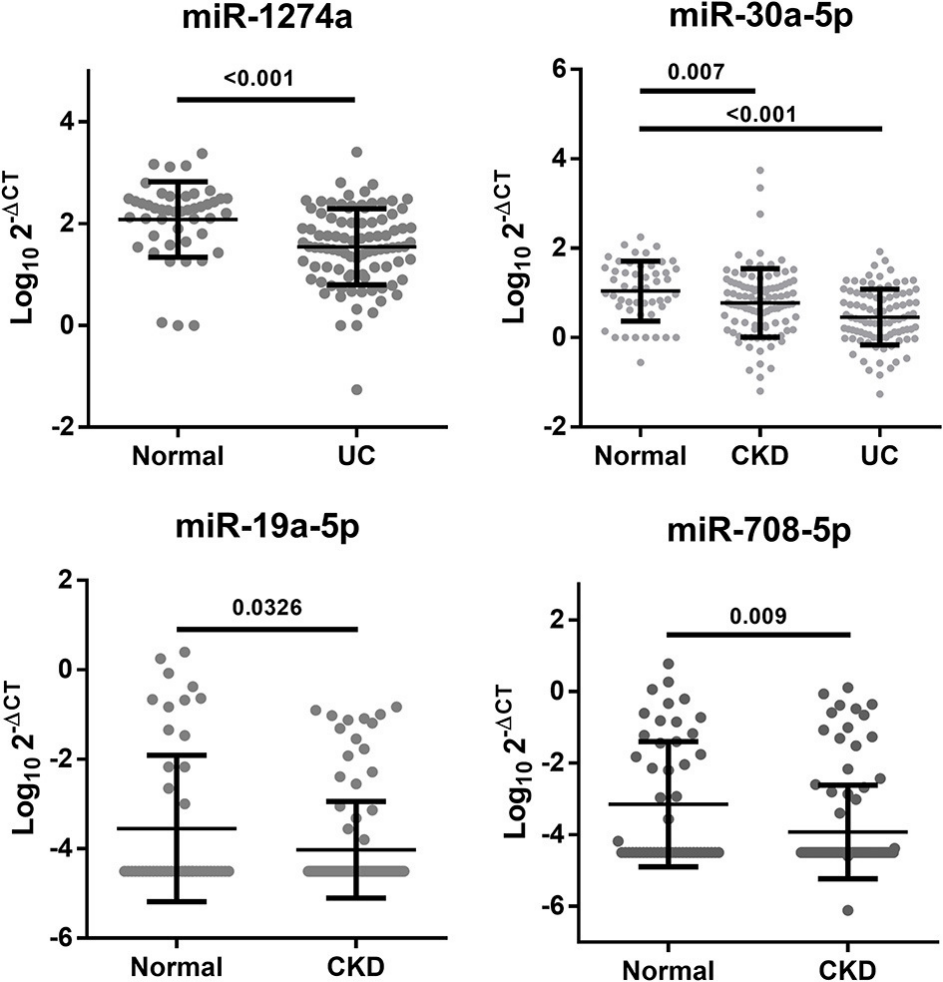
Significant changes in the urine miRNA expression levels between the healthy, CKD and CKD+UC groups. The Y axis presents the expression level (Log102-ΔCT). Healthy, healthy donors. CKD, patients with chronic kidney disease. UC, the urothelial carcinoma patients with CKD. The *p*-value was analyzed by Student's *t*-test for each miRNA.

Many studies have compared the different miRNA expression levels between the healthy group and patients with UC (28–30). Unlike previous studies, we tried to compare miRNA expression differences to identify UC from patients with CKD. To determine whether these candidate miRNAs from this study also have the potential to distinguish from the healthy group, we further collected 50 healthy cases to analyze the differences within the healthy, CKD and CKD + UC groups. miR-1274a and miR-30a-5p had significant differences between healthy cases and CKD + UC (*p* < 0.001). Interestingly, we found that three miRNAs, namely, miR-30a-5p, miR-19a-5p and miR-708-5p, not only can provide a reliable ability to distinguish patients who were CKD or CKD + UC (AUC = 0.64, 0.61, and 0.63, respectively) but also had significantly different expression levels between healthy subjects and CKD (*p* = 0.007, 0.0326, and 0.009, respectively) (Table 3; Figure 2).

**Table 3:** The area under the curve of candidate miRNAs in the training group.

**Urine sample**	**AUC**	**95% CI**
**A**		
miR-1274a	0.71	0.6090–0.8110
miR-19a-5p	0.61	0.4943–0.7169
miR-30a-5p	0.64	0.5342–0.7514
miR-708	0.63	0.6717–0.8611
**Plasma sample**	**AUC**	**95% CI**
**B**		
miR-155	0.65	0.5168–0.7773
miR-19b-1-5p	0.66	0.5327–0.7875
miR-210-3p	0.64	0.5107–0.7704
miR-636	0.61	0.4758–0.7431

*The receiver operating characteristic curve analysis for the candidate miRNAs is shown to distinguish patients with UC from those with CKD through urine samples (A) and plasma samples (B) (n = 100 and 70, respectively)*.

### miRNA Expression Levels as a Prognostic Marker of Bladder Cancer and Kidney Cancer

**Figure 3:**
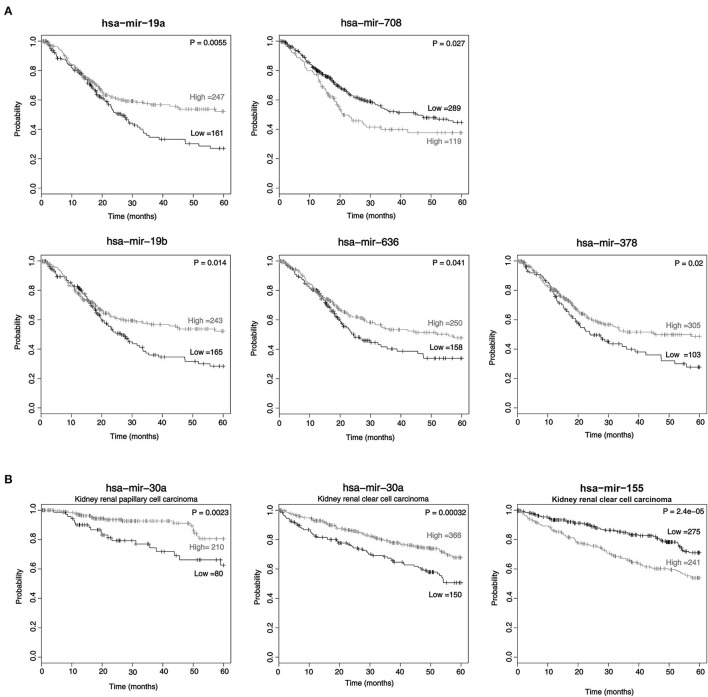
The expression of miRNAs in cancer tissue is associated with survival. The Kaplan-Meier survival curve of patients: low miRNA expression vs. high miRNA expression according to the automatic best cutoff from the database. The statistical significance of the difference in bladder cancer **(A)** and kidney renal papillary cell carcinoma and kidney renal clear cell carcinoma **(B)** are shown.

It has been known that miRNA expression is associated with cancer prognosis. Therefore, we investigated these 17 candidate miRNAs in a public database (http://kmplot.com) to analyze the association between newly identified miRNA expression levels and the 5-year survival rate by the Kaplan-Meier method. Among these miRNAs, lower expression levels of miR-19a, miR-19b, miR-636, and miR-378 and higher expression levels of miR-708-5p were associated with poor prognosis in BC (*p* = 0.0055, 0.014, 0.041, 0.02, and 0.027, respectively) (Figure 3A). In addition, lower expression of miR-30a and or higher miR-155 was associated with poor prognosis in urinary cancer, such as papillary cell carcinoma and clear cell renal cell carcinoma (Figure 3B).

### The Prediction Models to Predict UC for Patients With CKD

To develop a miRNA signature-based predicative model for UC of patients with all stages of CKD, receiver operating characteristic curve (ROC) analysis was performed. Seventeen candidate miRNA expression levels in urine or plasma from the training set samples were examined. The area under the receiver operating characteristic curve (AUC) is the most commonly used performance measure to indicate the discriminative ability of a prediction mode, and an AUC value higher than 0.6 could be a potential marker. Four miRNAs expressed in urine and four miRNAs expressed in plasma had AUC values above 0.6. The AUC values of miR-1274a, miR-19a-5p, miR-30a-5p and miR-708-5p in urine were 0.71, 0.61, 0.64, and 0.628, respectively (95% confidence intervals: 0.6113–0.8198, 0.5016–0.7304, 0.5980–0.8073, and 0.5136–0.7424, respectively) (Table 3A). In plasma samples, miR-155-5p, miR-19b-1-5p, miR-210 and miR-636 could be potential markers, and their AUC values were 0.65, 0.66, 0.64, and 0.61, respectively (95% confidence intervals: 0.5168–0.7773, 0.5327–0.7875, 0.5107–0.7704, and 0.4758–0.7431, respectively) (Table 3B). Interestingly, these miRNAs have been reported in previous studies to play key functions not only in BC but also in clear cell renal cell carcinoma (31–34).

**Figure 4:**
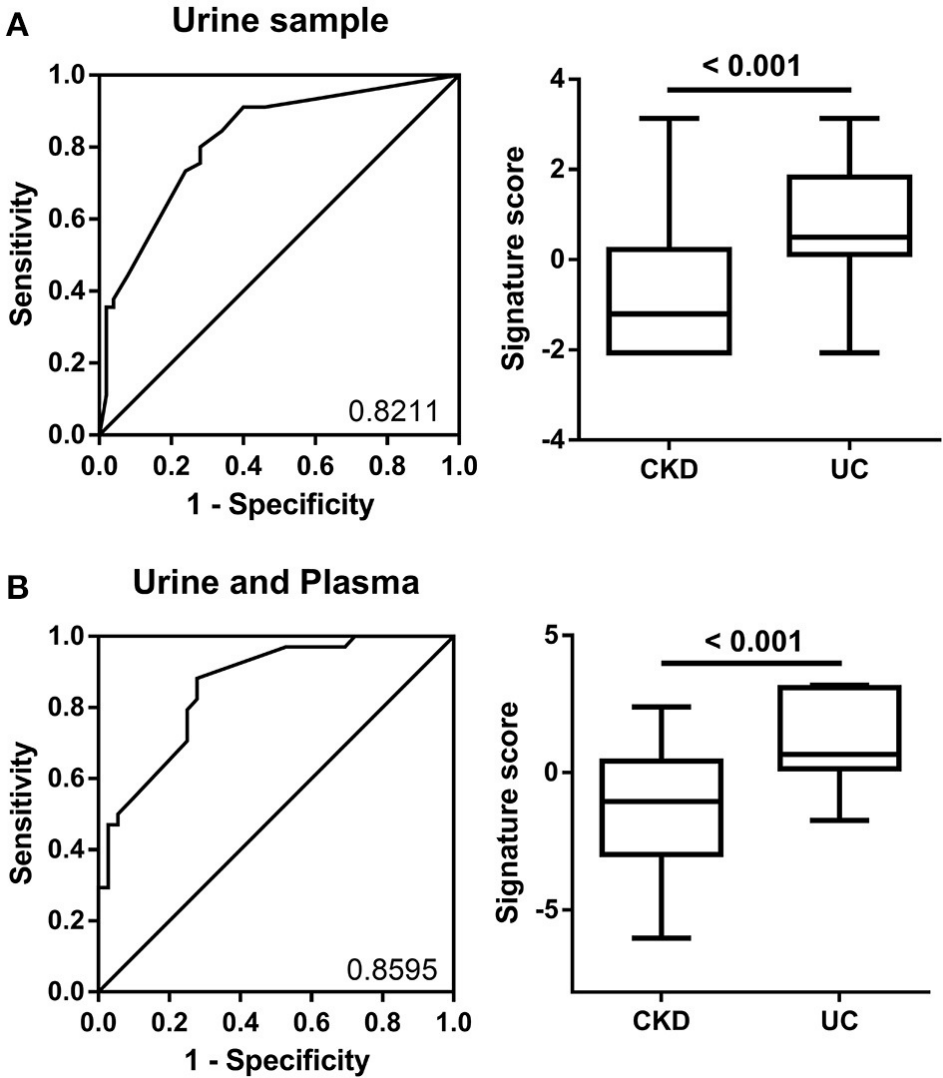
ROC curve analysis of miRNA combinations. **(A)** ROC curve analysis for miR-1274a, miR-19a-5p, miR-30a-5p, and miR-708-5p in urine was shown to distinguish patients with CKD + UC from those with CKD. **(B)** The ROC analysis for eight miRNAs (4 in urine-miR-1274a, miR-19a-5p, miR-30a-5p and miR-708-5p, and 4 in plasma-miR-155-5p, miR-19b-1-5p, miR-210 and miR-636) was shown to distinguish patients with CKD+UC from those with CKD. The box plots show the two prediction models distribution that combine the miRNA expression levels from the training group.

The combination of multiple factors compared to a single factor always presents more reliable prediction results for clinical classification. Therefore, we utilized multiple logistic regression calculation formulas to produce the prediction model combining different miRNA expression levels from the training group (Table 1). In the urine sample, the top four AUC values for miR-1274A, miR-30a-5p, miR-19b-3p, and miR-708-5p were combined and calculated together, and the AUC was 0.8211 (95% confidence interval: 0.7359–0.9063). We also validated this panel in the testing group, and the data from 200 patients show that the accuracy of the 4-miRNA signature in urine was 70%, based on the cutoff value > 0.483 (Figure 4A). Furthermore, we added another four miRNAs, namely, miR-155-5p, miR-19b-1-5p, miR-210 and miR-636, in plasma to increase the AUC value, and the AUC value increased up to 0.8507 (95% confidence interval: 0.7751–0.9439). The accuracy of the 8-miRNA signature was 72%, based on the cutoff value > −0.5940 (Figure 4B).

### Nomogram Construction Based on miRNAs Expression Signature

**Figure 5:**
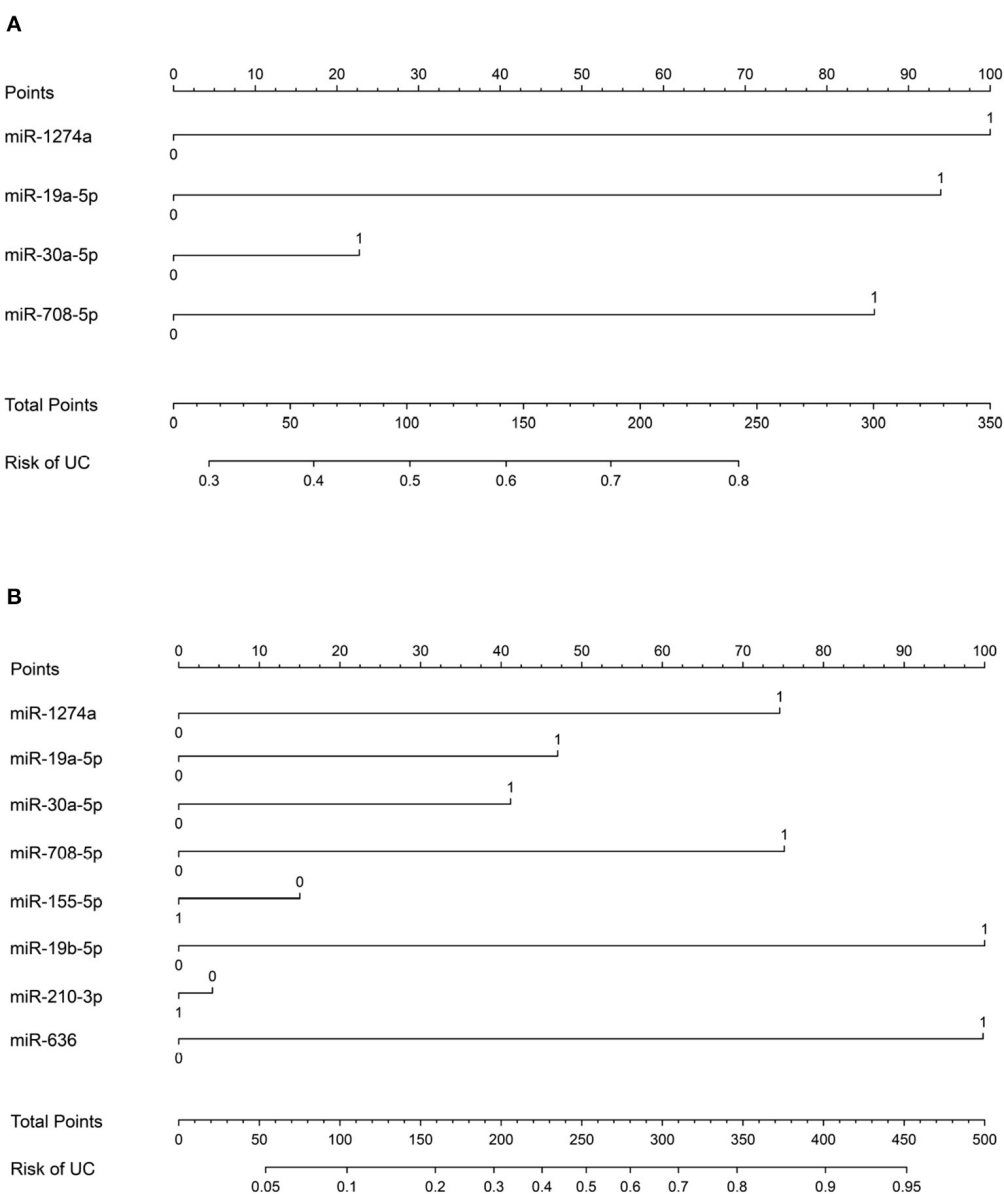
Nomogram for the diagnosis of urothelial cancer. **(A)** Nomogram plot from the points of four miRNA expressions in urine sample. **(B)** Nomogram plot from the points of eight miRNA expressions in urine and plasma sample.

In order to validate the risk of UC, a nomogram integrated miRNAs expression signature was established. The miRNA expression level was transformed to the points based on the cutoff value from the training group. The cutoff of miR-1274a, miR-19a-5p, miR-30a-5p and miR-708-5p were < 34.41, > 2.24^*^10^−4^, <3.798 and > 2.235^*^10^−7^, respectively. The AUC of the nomogram for urine samples were 0.7383 (*n* = 200, 95% confidence interval: to 0.6685–0.8080) (Figure 5A). Furthermore, the cutoff of miR-155-5p, miR-19b-5p, miR-210-3p, miR-378 and miR-636 were <1.21, <0.5107, <4.766, and <0.5722, respectively. The AUC of the nomogram for urine and plasma samples were 0.8096 (*n* = 138, 95% confidence interval: 0.7365–0.8827) (Figure 5B).

## Discussion

We found that the expression levels of miR-1274a and miR-30a-5p were significantly lower in CKD+UC compared with patients with CKD in the urine samples, but conversely, miR-19a-5p was significantly higher in CKD+UC patients (Figure 1A). High expression levels of miR-1274a have been demonstrated in clear cell renal cell carcinoma (ccRCC) compared with adjacent normal cells, which further induced cell apoptosis through the regulation of BMPR1B expression (34). The expression level of miR-30a-5p had significant decreased about 40% in the plasma samples of the BC patients when compared to the healthy, indicating lower expression of miR-30a-5p in urine due to the filtering on renal corpuscle (26). miR-30a-5p showed lower expression in UTUC compared with normal tissue, which was linked to decreased epithelial-to-mesenchymal transition (EMT) through regulation of the tight junction protein claudin-5 (33). Another study showed that miR-30a-5p expression was lower in muscle invasive BC and that overexpression of miR-30a-5p inhibited the malignancy of UC through Notch-1 gene regulation (32). In addition, compared with the healthy group, miR-19a (miR-19a-3p or miR-19a-5p) showed higher expression levels in the samples of BC such as cell lines, tissue and plasma (28).

Our results showed that the expression levels of miR-19b-1-5p, miR-378, miR-636 and miR-155-5p were significantly lower in CKD+UC plasma samples (Figure 1B). The data of hazard ratio showed that the miR-19b (miR-19b-3p or miR-19b-5p) expression level was highly correlated with the incidence of BC. Higher miR-19b (miR-19b-3p or miR-19b-5p) expression levels were found in ccRCC tissue, and miR-19b-3p promoted the malignancy of ccRCC through RhoB gene expression (35, 36). Lower miR-378 (miR-378-3p or miR-378-5p) expression levels were significantly linked to the high-risk group suffering from prostate cancer (37). miR-155-3p and miR-155-5p showed a higher expression level in the urine and tissue of patients with BC (27, 38, 39). It has been demonstrated that miR-155-5p is a key regulator that promotes BC growth through DMTF1 regulation.

Interestingly, our results revealed that miR-155-5p had significantly lower expression in plasma samples of patients with UC compared to patients with CKD. However, other reports showed that miR-155-3p and miR-155-5p had higher expression in the urine, plasma and tissues of patients with BC compared to the healthy population (24, 27, 38, 39). In addition, three studies indicated different expression levels of miR-378 (miR-378-3p or miR-378-5p) in RCC compared to the serum of healthy (40–42). Importantly, the statistical methods, including the calculation of expression levels and different internal controls, led to different results. The expression levels of miRNAs were inconsistent between cells and urine, possibly due to tissue specificity or the different functional effects between cellular and extracellular environments.

Our results showed that lower expression of miR-19a (miR-19a-3p or miR-19a-5p), miR-19b (miR-19b-3p or miR-19b-1-5p), miR-636 and miR-378 and higher expression of miR-708-5p were linked to the poor prognosis of patients with BC (Figure 3A). On the other hand, the group with lower miR-30a expression and higher miR-155 expression was linked to the poor prognosis of ccRCC (Figure 3B). Interestingly, a previous study showed that high miR-19a-3p expression was associated with poor prognosis of prostate cancer (43). Low expression of miR-19b-1-5p in tissue was linked to poor prognosis of BC, and low miR-19b-3p expression in patients suffering from prostate cancer also showed poor prognosis (24, 43). Poor prognosis was also found in the group with low miR-378 (miR-378-3p or miR-378-5p) expression in the plasma of RCC (41). In a previous study, miR-708-5p was reported in non-small cell lung cancer, ovarian cancer and stomach cancer (44–46).

## Conclusions

In this study, we aimed to establish predictive models of UC using miRNA expression levels in the urine and plasma. The prediction models and nomograms could be an ancillary diagnostic marker for patients with CKD, who are at high risk of developing UC. As far as we know, this is the first study to investigate UC in CKD patients by miRNA expression levels in their biofluids.

## Data Availability Statement

The original contributions presented in the study are included in the article/Supplementary Material, further inquiries can be directed to the corresponding author/s.

## Ethics Statement

The studies involving human participants were reviewed and approved by CMUH 102-REC2-043. The patients/participants provided their written informed consent to participate in this study.

## Author Contributions

C-CH and NM: conceptualization. A-LL, C-YC, and S-CL: methodology. A-LL, C-YC, NM, and C-CH: investigation and validation. C-LC, K-LW, H-CC, M-CW, C-CC, B-GH, and M-SW: resources. A-LL: writing—original draft. NM, C-LC, and C-CH: review and editing. NM, C-LC, and C-CH: funding acquisition. All authors contributed to the article and approved the submitted version.

## Funding

This work was supported by the following programs: Academia Sinica, Grant Numbers BM10701010023, BM10601010037, BM104010113, and BM103010089, NCU-Landseed International Chronic Disease Research Center, Grant Numbers NCU-LSH-108-A-005 and NCU-LSH-109-A-004, Ministry of Science and Technology, Grant Numbers MOST109-2628-B-008-001 and MOST 110-2823-8-008-002, and National Health Research Institutes, Grant Number NHRI-109BCCO-MF-202018-01.

## Conflict of Interest

The authors declare that the research was conducted in the absence of any commercial or financial relationships that could be construed as a potential conflict of interest.

## Publisher's Note

All claims expressed in this article are solely those of the authors and do not necessarily represent those of their affiliated organizations, or those of the publisher, the editors and the reviewers. Any product that may be evaluated in this article, or claim that may be made by its manufacturer, is not guaranteed or endorsed by the publisher.

## References

[1] Bray F Ferlay J Soerjomataram I Siegel RL Torre LA Jemal A. Global cancer statistics 2018: GLOBOCAN estimates of incidence and mortality worldwide for 36 cancers in 185 countries. CA Cancer J Clin. (2018) 68:394–424. 10.3322/caac.21492 30207593

[2] Kaseb H Aeddula NR. Cancer, Bladder. Treasure Island, FL: StatPearls (2019).

[3] Yang MH Chen KK Yen CC Wang WS Chang YH Huang WJ. Unusually high incidence of upper urinary tract urothelial carcinoma in Taiwan. Urology. (2002) 59:681–7. 10.1016/S0090-4295(02)01529-7 11992840

[4] Lowrance WT Ordonez J Udaltsova N Russo P Go AS. CKD and the risk of incident cancer. J Am Soc Nephrol. (2014) 25:2327–34. 10.1681/ASN.2013060604 24876115 PMC4178430

[5] Chen JS Lu CL Huang LC Shen CH Chen SC. Chronic kidney disease is associated with upper tract urothelial carcinoma: a nationwide population-based cohort study in Taiwan. Medicine. (2016) 95:e3255. 10.1097/MD.0000000000003255 27057873 PMC4998789

[6] Hsieh JJ Purdue MP Signoretti S Swanton C Albiges L Schmidinger M. Renal cell carcinoma. Nat Rev Dis Primers. (2017) 3:17009. 10.1038/nrdp.2017.9 28276433 PMC5936048

[7] Hatakeyama S Koie T Narita T Hosogoe S Yamamoto H Tobisawa Y. Renal function outcomes and risk factors for stage 3B chronic kidney disease after urinary diversion in patients with muscle invasive bladder cancer. PLoS ONE. (2016) 11:e0149544. 10.1371/journal.pone.0149544 26901860 PMC4763863

[8] Kodama H Hatakeyama S Fujita N Iwamura H Anan G Fukushi K. Preoperative chronic kidney disease predicts poor oncological outcomes after radical nephroureterectomy in patients with upper urinary tract urothelial carcinoma. Oncotarget. (2017) 8:83183–94. 10.18632/oncotarget.20554 29137333 PMC5669959

[9] Lotan Y Elias K Svatek RS Bagrodia A Nuss G Moran B. Bladder cancer screening in a high risk asymptomatic population using a point of care urine based protein tumor marker. J Urol. (2009) 182:52–7. 10.1016/j.juro.2009.02.142 19450825

[10] Xi Z Linlin M Ye T. Human epididymis protein 4 is a biomarker for transitional cell carcinoma in the urinary system. J Clin Lab Anal. (2009) 23:357–61. 10.1002/jcla.20329 19927341 PMC6649142

[11] Manvar AM Wallen EM Pruthi RS Nielsen ME. Prognostic value of CA 125 in transitional cell carcinoma of the bladder. Expert Rev Anticancer Ther. (2010) 10:1877–81. 10.1586/era.10.186 21110754

[12] Ahmadi H Djaladat H Cai J Miranda G Daneshmand S. Precystectomy serum levels of carbohydrate antigen 19-9, carbohydrate antigen 125, and carcinoembryonic antigen: prognostic value in invasive urothelial carcinoma of the bladder. Urol Oncol. (2014) 32:648–56. 10.1016/j.urolonc.2014.01.019 24680660

[13] Chou CY Shu KH Chen HC Wang MC Chang CC Hsu BG. Development and validation of a nomogram for urothelial cancer in patients with chronic kidney disease. Sci Rep. (2019) 9:3473. 10.1038/s41598-019-40276-4 30837585 PMC6401318

[14] Sanli O Dobruch J Knowles MA Burger M Alemozaffar M Nielsen ME. Bladder cancer. Nat Rev Dis Primers. (2017) 3:17022. 10.1038/nrdp.2017.22 28406148

[15] Gaggl M Hofer M Weidner S Kleinert J Fauler G Wallner M. Interfering parameters in the determination of urinary globotriaosylceramide (Gb3) in patients with chronic kidney disease. J Nephrol. (2015) 28:679–89. 10.1007/s40620-015-0193-1 25857295

[16] Fendler A Stephan C Yousef GM Kristiansen G Jung K. The translational potential of microRNAs as biofluid markers of urological tumours. Nat Rev Urol. (2016) 13:734–52. 10.1038/nrurol.2016.193 27804986

[17] Mytsyk Y Dosenko V Skrzypczyk MA Borys Y Diychuk Y Kucher A. Potential clinical applications of microRNAs as biomarkers for renal cell carcinoma. Cent European J Urol. (2018) 71:295–303. 10.5173/ceju.2018.1618 30386650 PMC6202627

[18] Andersen GB Tost J. Circulating miRNAs as biomarker in cancer. Recent Results Cancer Res. (2020) 215:277–98. 10.1007/978-3-030-26439-0_15 31605235

[19] Springer SU Chen CH Rodriguez Pena MDC Li L Douville C Wang Y. Non-invasive detection of urothelial cancer through the analysis of driver gene mutations and aneuploidy. Elife. (2018) 7:e32143. 10.1101/203976 29557778 PMC5860864

[20] Blanca A Sanchez-Gonzalez A Requena MJ Carrasco-Valiente J Gomez-Gomez E Cheng L. Expression of miR-100 and miR-138 as prognostic biomarkers in non-muscle-invasive bladder cancer. APMIS. (2019) 127:545–53. 10.1111/apm.12973 31231851

[21] Gullu Amuran G Tinay I Filinte D Ilgin C Peker Eyuboglu I Akkiprik M. Urinary micro-RNA expressions and protein concentrations may differentiate bladder cancer patients from healthy controls. Int Urol Nephrol. (2019) 52:461–8. 10.1007/s11255-019-02328-6 31679136

[22] Lin GB Zhang CM Chen XY Wang JW Chen S Tang SY. Identification of circulating miRNAs as novel prognostic biomarkers for bladder cancer. Math Biosci Eng. (2019) 17:834–44. 10.3934/mbe.2020044 31731380

[23] Usuba W Urabe F Yamamoto Y Matsuzaki J Sasaki H Ichikawa M. Circulating miRNA panels for specific and early detection in bladder cancer. Cancer Sci. (2019) 110:408–19. 10.1111/cas.13856 30382619 PMC6317958

[24] Chen CL Lin CH Li AL Huang CC Shen BY Chiang YR. Plasma miRNA profile is a biomarker associated with urothelial carcinoma in chronic hemodialysis patients. Am J Physiol Renal Physiol. (2019) 316:F1094–102. 10.1152/ajprenal.00014.2019 30892932

[25] Li AL Chung TS Chan YN Chen CL Lin SC Chiang YR. microRNA expression pattern as an ancillary prognostic signature for radiotherapy. J Transl Med. (2018) 16:341. 10.1186/s12967-018-1711-4 30518388 PMC6282371

[26] Jiang X Du L Wang L Li J Liu Y Zheng G. Serum microRNA expression signatures identified from genome-wide microRNA profiling serve as novel noninvasive biomarkers for diagnosis and recurrence of bladder cancer. Int J Cancer. (2015) 136:854–62. 10.1002/ijc.29041 24961907

[27] Wang G Chan ES Kwan BC Li PK Yip SK Szeto CC. Expression of microRNAs in the urine of patients with bladder cancer. Clin Genitourin Cancer. (2012) 10:106–13. 10.1016/j.clgc.2012.01.001 22386240

[28] Feng Y Liu J Kang Y He Y Liang B Yang P. miR-19a acts as an oncogenic microRNA and is up-regulated in bladder cancer. J Exp Clin Cancer Res. (2014) 33:67. 10.1186/s13046-014-0067-8 25107371 PMC4237814

[29] Du M Shi D Yuan L Li P Chu H Qin C. Circulating miR-497 and miR-663b in plasma are potential novel biomarkers for bladder cancer. Sci Rep. (2015) 5:10437. 10.1038/srep10437 26014226 PMC4444850

[30] Long JD Sullivan TB Humphrey J Logvinenko T Summerhayes KA Kozinn S. A non-invasive miRNA based assay to detect bladder cancer in cell-free urine. Am J Transl Res. (2015) 7:2500–9. 26807194 PMC4697726

[31] Song T Zhang X Zhang L Dong J Cai W Gao J. miR-708 promotes the development of bladder carcinoma via direct repression of Caspase-2. J Cancer Res Clin Oncol. (2013) 139:1189–98. 10.1007/s00432-013-1392-6 23568547 PMC11824749

[32] Zhang C Ma X Du J Yao Z Shi T Ai Q. MicroRNA-30a as a prognostic factor in urothelial carcinoma of bladder inhibits cellular malignancy by antagonising Notch1. BJU Int. (2016) 118:578–89. 10.1111/bju.13407 26775686

[33] Chung YH Li SC Kao YH Luo HL Cheng YT Lin PR. MiR-30a-5p inhibits epithelial-to-mesenchymal transition and upregulates expression of tight junction protein claudin-5 in human upper tract urothelial carcinoma cells. Int J Mol Sci. (2017) 18:1826. 10.3390/ijms18081826 28829370 PMC5578210

[34] Yoshino H Yonezawa T Yonemori M Miyamoto K Sakaguchi T Sugita S. Downregulation of microRNA-1274a induces cell apoptosis through regulation of BMPR1B in clear cell renal cell carcinoma. Oncol Rep. (2018) 39:173–81. 10.3892/or.2017.6098 29192325

[35] Niu S Ma X Zhang Y Liu YN Chen X Gong H. MicroRNA-19a and microRNA-19b promote the malignancy of clear cell renal cell carcinoma through targeting the tumor suppressor RhoB. PLoS ONE. (2018) 13:e0192790. 10.1371/journal.pone.0192790 29474434 PMC5825063

[36] Yin XH Jin YH Cao Y Wong Y Weng H Sun C. Development of a 21-miRNA signature associated with the prognosis of patients with bladder cancer. Front Oncol. (2019) 9:729. 10.3389/fonc.2019.00729 31448232 PMC6692470

[37] Nguyen HC Xie W Yang M Hsieh CL Drouin S Lee GS. Expression differences of circulating microRNAs in metastatic castration resistant prostate cancer and low-risk, localized prostate cancer. Prostate. (2013) 73:346–54. 10.1002/pros.22572 22887127 PMC3980954

[38] Peng Y Dong W Lin TX Zhong GZ Liao B Wang B. MicroRNA-155 promotes bladder cancer growth by repressing the tumor suppressor DMTF1. Oncotarget. (2015) 6:16043–58. 10.18632/oncotarget.3755 25965824 PMC4599255

[39] Zhang X Zhang Y Liu X Fang A Wang J Yang Y. Direct quantitative detection for cell-free miR-155 in urine: a potential role in diagnosis and prognosis for non-muscle invasive bladder cancer. Oncotarget. (2016) 7:3255–66. 10.18632/oncotarget.6487 26657502 PMC4823104

[40] Redova M Poprach A Nekvindova J Iliev R Radova L Lakomy R. Circulating miR-378 and miR-451 in serum are potential biomarkers for renal cell carcinoma. J Transl Med. (2012) 10:55. 10.1186/1479-5876-10-55 22440013 PMC3340316

[41] Fedorko M Stanik M Iliev R Redova-Lojova M Machackova T Svoboda M. Combination of MiR-378 and MiR-210 serum levels enables sensitive detection of renal cell carcinoma. Int J Mol Sci. (2015) 16:23382–9. 10.3390/ijms161023382 26426010 PMC4632704

[42] Wang C Hu J Lu M Gu H Zhou X Chen X. A panel of five serum miRNAs as a potential diagnostic tool for early-stage renal cell carcinoma. Sci Rep. (2015) 5:7610. 10.1038/srep07610 25556603 PMC5154588

[43] Stuopelyte K Daniunaite K Jankevicius F Jarmalaite S. Detection of miRNAs in urine of prostate cancer patients. Medicina. (2016) 52:116–24. 10.1016/j.medici.2016.02.007 27170485

[44] Jang JS Jeon HS Sun Z Aubry MC Tang H Park CH. Increased miR-708 expression in NSCLC and its association with poor survival in lung adenocarcinoma from never smokers. Clin Cancer Res. (2012) 18:3658–67. 10.1158/1078-0432.CCR-11-2857 22573352 PMC3616503

[45] Lin KT Yeh YM Chuang CM Yang SY Chang JW Sun SP. Glucocorticoids mediate induction of microRNA-708 to suppress ovarian cancer metastasis through targeting Rap1B. Nat Commun. (2015) 6:5917. 10.1038/ncomms6917 25569036 PMC4354140

[46] Liang L Zhang L Cui D Yang D. Identification of the key miRNAs associated with survival time in stomach adenocarcinoma. Oncol Lett. (2017) 14:4563–72. 10.3892/ol.2017.6792 29085454 PMC5649651

